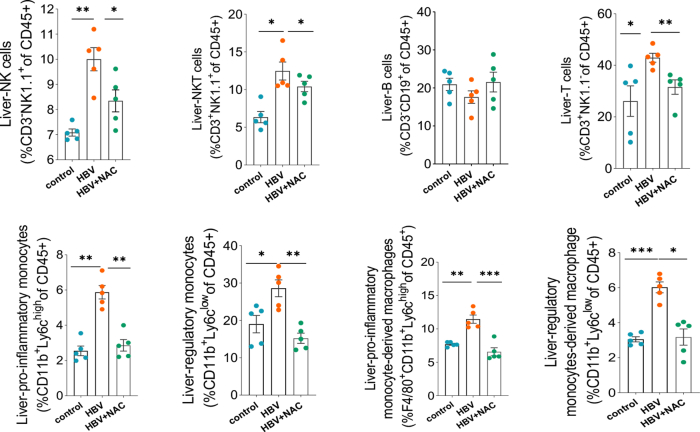# Activation of the Jak2/Stat3 pathway by ROS-dependent signaling cascades initiates hepatitis B virus-induced hepatic inflammatory responses

**DOI:** 10.1016/j.gendis.2025.101857

**Published:** 2025-09-23

**Authors:** Rui Song, Shasha Yu, Xueyan Chen, Ning Ling, Dachuan Cai, Hong Ren, Min Chen

**Affiliations:** Department of Infectious Diseases, Institute for Viral Hepatitis, Key Laboratory of Molecular Biology for Infectious Diseases (Ministry of Education), Second Affiliated Hospital of Chongqing Medical University, Chongqing 400010, China

**Keywords:** HBV, Liver inflammation, Mitochondrial respiration, ROS, Stat3

## Abstract

Chronic liver necroinflammation induced by hepatitis B virus (HBV) infection plays a major causative role in the development of end-stage liver diseases; however, mechanisms contributing to its initiation remain unclear. Analysis of the hepatic transcriptome from HBV-replication mice or HBV-infected patients revealed that significantly down-regulated mitochondrial oxidative phosphorylation function was the salient transcriptional feature at the early stage of liver inflammation compared with the stage without liver inflammation. In cell models, persistent HBV replication-induced progressive impairment of mitochondrial respiration resulted in increased reactive oxygen species (ROS) levels. We further discovered that HBV replication-induced ROS accumulation was essential for the up-regulation of nuclear factor erythroid 2-related factor 2 (Nrf2)-associated interleukin (IL)-6/IL-8 production, mediating the activation of Janus kinase 2 (Jak2)/signal transducer and activator of transcription (Stat3) signaling, and then the expression of downstream inflammatory genes. These observations were also identified in HBV-replication mice at the early stage of liver inflammation, which exhibited elevated hepatic oxidative stress, Nrf2 expression, IL-6 and IL-8 production, and Jak2/Stat3 activation, alongside hepatic inflammatory cell infiltration. *In vivo*, ROS scavenging with N-acetylcysteine (NAC) mitigated these effects. Our findings underscore the critical role of ROS-dependent Jak2/Stat3 pathway activation in the occurrence of HBV-induced liver inflammation, providing new insights into the pathogenesis of chronic hepatitis B.

## Introduction

Currently, hepatitis virus B (HBV) infection is still a major public health issue, with an estimated 296 million people being chronically infected and approximately 1.1 million deaths globally in 2022, mainly due to HBV-related cirrhosis and liver cancer.^1^ Most HBV infections in immunocompetent adults spontaneously resolve, and less than 5% of cases become chronic, whereas up to 90% of all chronic HBV infections result from infections acquired in infancy and early childhood.^1^ The natural history of chronic HBV infection (chronic hepatitis B, CHB) includes four distinct phases, each characterized by varying levels of viral replication, viral antigen expression, and inflammation in the liver. The first phase is called the “immune-tolerant phase (IT phase)”, typically observed after the perinatally or early childhood-acquired infection and characterized by high viral replication, the presence of hepatitis B e-antigen (HBeAg), normal serum alanine aminotransferase (ALT) levels, and minimal or no liver inflammation or fibrosis on biopsy. During adolescence or adulthood, these patients in the immune-tolerant phase usually progress into the second phase, the “immune-active phase (IA phase)”, which is marked by fluctuating high serum ALT levels, decreased levels of HBV DNA and HBeAg, and moderate-to-severe liver inflammation or fibrosis.^2^, ^3^, ^4^ However, the mechanism triggering this transition from the immune-tolerant phase to the immune-active phase still remains unclear.

It was previously thought that increased numbers or activity of HBV-specific T cells with age was the key factor mediating the transition from the immune-tolerant phase to the immune-active phase.^5^ However, this hypothesis was substantially challenged by recent studies on pediatric patients in the immune-tolerant phase, who could mount HBV-specific T-cell responses, although these responses were less compromised than those of adult patients in the immune-active phase.^6^^,^^7^ Therefore, an alternative interpretation was proposed that elevated hepatic non-specific pro-inflammatory responses were responsible for this event.^8^ This viewpoint was supported by the latest studies on liver transcriptome or single-cell transcriptome of liver-infiltrating immune cells from CHB patients during the immune-tolerant and the immune-active phase. These results demonstrated that non-specific immune or inflammation-related gene expression or myeloid cell infiltration were remarkably enhanced in both patients in the immune-tolerant and the immune-active phase when compared with healthy controls.^9^^,^^10^ However, due to the difficulties in the longitudinal collection of liver tissue of the patients in the immune-tolerant phase, the molecular mechanism underlying the transition from the immune-tolerant phase to the immune-active phase remains unclear yet.

In our previous study, we explored this mechanism by dynamically analyzing the liver transcriptome of an HBV transgenic mouse (HBV-Tg) model with high levels of viral replication and spontaneous liver inflammation. Our results revealed that the activation of signal transducers and activators of transcription 3 (Stat3) triggered the expression of inflammation-related genes and governed the early stage of HBV-induced hepatic inflammation.^11^ These findings provide valuable insights for further investigation of the mechanism governing upstream regulators of Stat3 activation.

Notably, some liver transcriptome-based studies have revealed that several metabolic pathways, such as oxidative phosphorylation (OXPHOS), fatty acid metabolism, and bile acid metabolism, are down-regulated in CHB patients with a relatively higher expression of immune genes and infiltration of immune cells.^9^ As we know, mitochondria serve as the primary site for oxidative phosphorylation and reactive oxygen species (ROS) production and are the central hubs for regulating metabolic pathways and controlling cellular signal transduction.^12^^,^^13^ Therefore, these evidences suggest that mitochondrial dysfunction in chronic HBV infections might play a vital role in these metabolic changes and inflammatory infiltration. Recent research has indicated that HBV X protein (HBx) overexpression in experimental models can induce alterations in the mitochondrial structure and function and cause oxidative stress.^14^, ^15^, ^16^ Furthermore, mitochondrial oxidative stress, mtDNA damage, and associated alterations in mitochondrial function and dynamics were observed in patients with HBV advanced fibrosis or cirrhosis.^17^^,^^18^ However, whether mitochondrial oxidative stress acts as a key initiator of HBV-induced inflammation that contributes to the transition from the immune-tolerant to the immune-active phase has not yet been discovered.

Therefore, this study focuses on HBV replication-induced changes in mitochondrial OXPHOS and ROS production, and the roles of mitochondrial oxidative stress in Stat3 activation and liver inflammation occurrence. These results would extend our previous findings, demonstrating the upstream events of Stat3 activation in HBV-induced liver inflammation, which bring some new insights into the intricate molecular mechanisms of the transition from the immune-tolerant to the immune-active phase in chronic HBV infection.

## Materials and methods

### Cell lines and treatment

Human hepatoma cell line HepG2, HepG2.2.15, and HepAD38 (derived from HepG2 cells with stable HBV production) were cultured in Dulbecco’s Modified Eagle Medium (DMEM) containing 10% fetal bovine serum (FBS), with penicillin (100 U/mL) and streptomycin (0.1 mg/mL; P1410, Solarbio, China) at 37 °C in 5% CO_2_ cell incubator. In addition, HepG2.2.15 and HepAD38 cells were cultured in the presence of G418 (400 μg/mL; IG0010, Solarbio, China). Mito-TEMPO (HY-112879, MedChemExpress, USA) or H_2_O_2_ (HY-112879, BOSTER, China) was introduced to decrease or elevate ROS levels in these cell lines. Entecavir (20 μM; HY-13623, MedChemExpress, USA) was utilized to inhibit HBV replication in HepG2.2.15 and HepAD38 cells. The cells were seeded into 6-well plates and incubated overnight under standard culture conditions. On the following day, the culture medium was replaced with fresh medium containing entecavir (20 μM). The entecavir-containing medium was refreshed daily. After 3 days of treatment, the cells were collected for subsequent experiments.

### Establishment of a persistent HBV replication mouse model and treatment

Male C57BL/6J mice, aged 5–6 weeks, were obtained from the experimental animal center of Chongqing Medical University (Chongqing, China). The pAAV/HBV1.2 plasmid, containing a 1.2-fold over-length HBV genome, was kindly provided by Professor Pei-Jer Chen and preserved in our institute. To establish an HBV-replicating mouse model, 6 μg of pAAV/HBV1.2 plasmid was hydrodynamically injected into the mouse tail vein in a volume of phosphate-buffered saline (PBS) equivalent to 8%–10% of the mouse body weight. Serum from both HBV-replicating mice (pAAV/HBV1.2 plasmid) and normal controls (PBS) was collected at 2, 3, 4, and 5 weeks after injection for the detection of levels of HBV DNA, HBsAg, HBeAg, and ALT. At the same time, liver tissue samples were also collected for analysis of histopathology, ROS level, immune cell infiltration, and protein expression.

Additionally, N-acetyl-L-cysteine (NAC; HY-B0215, MedChemExpress, USA), an effective ROS scavenger, was applied to reduce the hepatic ROS in HBV mice. Two weeks after hydrodynamical injection, HBV replication mice were injected intraperitoneally every day with either NAC (300 mg/kg at 200 μL saline; HBV-NAC group) or saline solution (HBV group) for another two weeks. Four weeks after hydrodynamical injection, mice of the normal control, HBV-NAC, and HBV groups were sacrificed, and serum and liver tissue samples were collected for subsequent experiments.

Five to seven mice were examined in each group. All mouse handling methods and experimental procedures were approved by the Ethics Committee of Chongqing Medical University.

### Mitochondrial OXPHOS-related gene profiles by analysis of hepatic transcriptome from HBV-replicating mice and chronic HBV-infected patients (GSE83148)

We have previously reported the analysis results of comparing the hepatic transcriptome data from HBV-Tg mice to normal control mice, or chronic HBV-infected patients (GSE83148) to healthy controls.^11^ By consensus of the clustering analysis of hepatic transcriptome data, the unique early stage of HBV-induced hepatic inflammation was identified in 3-month-old HBV-Tg mice and certain chronic HBV-infected patients during the early immune-active phase.^11^ In this study, to explore the potential mechanism of the early stage of HBV-induced hepatic inflammation, we further compared the hepatic transcriptome profiles between the stage of no obvious hepatic inflammation and the early stage of hepatic inflammation in HBV transgenic mice (1-month-old *vs*. 3-month-old) or the patients with chronic HBV-infection (the immune-tolerant phase *vs*. the early immune-active phase) by Gene Set Enrichment Analysis (GSEA).

### Measurement of mitochondrial function

#### Oxygen consumption rate detection

Oxygen consumption rates were analyzed in 96-well plates using a Seahorse Cell Mito Stress Test Kit (#103010-100) by an XF96 analyzer (Agilent Technologies, USA), under various treatment conditions. HepG2, HepG2.2.15, or HepAD38 cells were seeded 2 × 10^5^ cells per well in 100 μL of DMEM supplemented with 10% fetal bovine serum for cell culture. After 72 h of incubation, the cells were washed with Seahorse RPMI medium and incubated at 37 °C without CO_2_ for 60 min. During the detection process, oligomycin (2.0 μM), carbonyl cyanide-4 (trifluoromethoxy) phenylhydrazone (FCCP, 1.0 μM), and rotenone/antimycin A (0.5 μM) were injected at the indicated times, and then the mitochondrial stress test program was run according to a predetermined template. Cell count standardization was performed after the experiment.

### Mitochondrial membrane potential

The JC-1 assay kit (C2003S, Beyotime, China) was used to detect mitochondrial membrane potential. Briefly, 5 × 10^5^ HepG2, HepG2.2.15, or HepAD38 cells were stained with 500 μL of diluted JC-1 working solution in an Eppendorf tube at 37 °C in the dark for 20 min. Following incubation, the cells were washed twice with JC-1 staining buffer and resuspended in 200 μL of PBS containing 1% fetal bovine serum for flow cytometry analysis.

### ATP content

The intracellular ATP content was detected using the ATP Content Assay Kit (BC0305, Solarbio, China). Briefly, 5 × 10^5^ cells were harvested in 100 μL assay buffer for lysate preparation via ultrasonic crushing. After centrifugation, the supernatant was transferred into another Eppendorf tube, mixed with 500 μL of chloroform, and shaken thoroughly. Finally, the supernatant was collected for the determination of ATP content according to the manufacturer’s instructions.

### Activity of mitochondrial complexes I and Ⅲ

The activity of mitochondrial complexes I and Ⅲ was detected using the mitochondrial complex I activity assay kit (BC0515, Solarbio, China) and mitochondrial complex III activity assay kit (BC3245, Solarbio, China), respectively. According to the manufacturer’s instructions, a total of 1 × 10^6^ cells were harvested and homogenized in 200 μL of lysis buffer. Subsequently, after 10 min of centrifugation at 600 *g* at 4 °C, the supernatant was discarded, and the precipitation was mixed with 200 μL of extraction solution and then crushed by ultrasonic waves to determine mitochondrial complex I or complex III enzyme activity.

### Measurement of ROS in cell lines and liver tissue

The levels of total cellular or mitochondrial-specific ROS were detected with DCFH-DA Kit (S0033S, Beyotime, China) or MitoSOX Green Kit (M36005, Invitrogen, USA). According to the manufacturer’s instructions, 5 × 10^5^ cells were harvested, mixed with 500 μL of DCFH-DA working solution (10 μM) or Mitosox Green working solution (5 μM), and incubated at 37 °C in the dark for 30 min. Then, the cells were washed twice with PBS, resuspended in 200 μL of PBS, and analyzed by flow cytometry.

The ROS level in the tissues was detected by DCFH-DA staining of frozen sections. Fresh liver tissues were rapidly frozen in liquid nitrogen and cut into 6–8 μm sections. These sections were stained with DCFH-DA (10 μM) at 37 °C in the dark for 30 min. After being washed twice, the nuclei of cells were stained with a Hoechst 33342 kit. Finally, the sections were sealed with an anti-fluorescence quenching agent (S2100, Solarbio, China) and observed under a fluorescence microscope.

### Measurement of reduced glutathione (GSH) and oxidized glutathione (GSSG)

The content of GSH or GSSG was measured by the GSH content assay kit (BC1175, Solarbio, China) and the GSSG content assay kit (BC1185, Solarbio, China), respectively. In brief, approximately 0.1 g of fresh liver tissue was homogenized in 1 mL of extract solution. After centrifugation at 800 *g* at 4 °C for 10 min, the supernatant was collected for GSH or GSSG detection in accordance with the instructions. The results were expressed as the GSH or GSSG content (μg) per gram of fresh liver tissue.

### Real-time quantitative PCR

TRIzol reagent (15596026CN, Invitrogen, USA) was applied to the lysed cells and liver tissues. RNA extraction was conducted using the Direct-zol™ RNA Miniprep kit (R2052, ZYMO RESEARCH, USA) according to the manufacturer’s instructions. Subsequently, complementary DNA (cDNA) was synthesized from approximately 1 μg RNA using the PrimeScriptTM RT reagent kit (RR047A, Takara, Japan). Gene expression was quantified through real-time PCR using TB Green Premix Ex Taq II Kit (RR820A, Takara, Japan), and the 2^–ΔΔCT^ method was employed for comparing the gene expression levels with normalization against β-actin mRNA. Three replicates were set for each PCR run, and their average values were used for relative mRNA expression. The primer pairs used in the study are listed in Table 1, Table 2.

**Table 1:** Primers used in the quantitative real-time PCR analysis (*Homo sapiens*).

Genes	Forward Primer Sequence (5′3′)	Reverse Primer Sequence (5′3′)
β-actin	AAGTGTGACGTTGACATCCG	GATCCACATCTGCTGGAAGG
Saal	CATTTGTTCACGAGGCTTTCC	GTTTTTCCAGTTAGCTTCCTTCATGT
S100a9	GACACCCTGACACCCTGAG	TGAGGGCTTCATTTCTCTTCTC
Icam1	GGGAATGTCACCAGGAATGT	GCACCAGAATGATTATAGTCCA
Socs3	TTCAGCTCCAAGAGCGAGTA	CGGAGTAGATGTAATATGGCTC
IL-6	GTAGCCGCCCCACACAGA	CATGTCTCCTTTCTCAGGGCTG
IFN-α	CCTTCCAAGATCACTGTTACCT	TTCTGCTCTGACCACCTCCC
IFN-γ	GGAGGAACTGGCAAAAGGAT	TTCAAGACTTCAAAGAGTCTGAGG
IL-8	CACCGGAAGGAACCATCTCA	AGAGCCACGGCCAGCTT
IL-10	GCCTAACATGCTTCGAGATC	TGATGTCTGGGTCTTGGTTC

**Table 2:** Primers used in the quantitative real-time PCR analysis (*Mus musculus*).

Genes	Forward Primer Sequence (5′3′)	Reverse Primer Sequence (5′3′)
β-actin	AGAAGATCTGGCACCACACC	TACGACCAGAGGCATACAGG
Saal	ACATGAAGGAAGCTGGCTGG	ATGTCTGTTGGCTTCCTGGTCA
S100a9	AGATGGCCAACAAAGCACCT	TAGACTTGGTTGGGCAGCTG
Icam1	GGGAATGTCACCAGGAATGT	GCACCAGAATGATTATAGTCCA
Socs3	TTCAGCTCCAAGAGCGAGTA	CGGAGTAGATGTAATATGGCTC
IL-6	TGTGTGAAAGCAGCAAAGA	ACCAGGCAAGTCTCCTCA
IL-8	GTGCTGTGTTGAATTACGGA	TTGACTGTGGAGTTTTGGC

### Western blotting

The total protein of cells or liver tissues was extracted using the Whole Cell Lysis Assay (KGP250, KeyGen Biotech, China) and quantified by the BCA Protein Quantitation Assay (KGPBCA, KeyGen Biotech, China). The sample proteins were separated by SDS-PAGE and transferred onto PVDF membranes (Millipore Corporation, USA). After being blocked with 5% skimmed milk solution, the membranes were incubated at 4 °C overnight with primary antibodies against mouse anti-Stat3 (ab119352, Abcam, USA), rabbit anti-p-Stat3 (phospho Y705; ab76315, Abcam, USA), rabbit anti-Jak2 (A19629, ABclonal, China), rabbit anti-p-Jak2 (32101, Abcam, USA), rabbit anti-Nrf2 (ab137550, Abcam USA), rabbit anti-IL-6 (A22222, ABclonal, China), and rabbit anti-IL-8 (A24736, ABclonal, China). The mouse anti-β-actin (AC004, ABclonal, China) was used as the internal control. Washed with Tris-buffered saline and Tween 20 (TBST) solution, the membranes were then incubated with horseradish peroxidase-linked anti-mouse or anti-rabbit IgG (7074P2 or 7076P2, CST, USA) at room temperature for 2 h. Protein bands were developed with WesternBright™ ECL (K-12045-D50, Advansta, USA) and detected by ChemicDoc™ MP Imaging System (Bio-Rad, USA).

### Evaluation of the levels of HBV DNA, HBsAg, HBeAg, and ALT in the serum of mice

Serum ALT levels were measured using the HITACHI Clinical Analyzer 7600 (Hitachi, Japan). The chemiluminescence microparticle immunoassay (CMIA) method was employed to quantify the HBsAg and HBeAg levels with Alinity HBsAg and HBeAg detection kits (Abbott Laboratories, Illinois, USA). The quantification of HBV-DNA was carried out according to the manufacturer’s instructions using real-time quantitative PCR with the COBAS AmpliPrep/COBAS TaqMan HBV Test Kit (v2.0) (Roche Diagnostic Systems, USA).

### Assessment of IL-6 and IL-8 levels

The levels of IL-6 and IL-8 in the serum of pAAV/HBV1.2 and control mice were quantified with ELISA kits (SEKM-0007 or SEKM-0046, Solarbio, China).

### Hematoxylin-eosin staining

The liver tissues from pAAV/HBV1.2 and control mice were fixed in 4% paraformaldehyde at room temperature for 24 h, dehydrated with an ethanol gradient, embedded in paraffin, and sectioned at 6–8 μm thickness. The paraffin slides were then deparaffinized, rehydrated with graded alcohols, and stained for histological examination using a hematoxylin-eosin stain kit (BC1185, Solarbio, China). Digital images were created and analyzed by a Digital Pathology System (3DHISTECH, Hungary) at a magnification of 200 ×. The Ishak scoring system was applied to assess the severity of liver inflammation and fibrosis.

### Intrahepatic leukocyte isolation

Briefly, the liver tissues of pAAV/HBV1.2 and control mice were thoroughly disaggregated mechanically into a cell suspension using a 200 metal mesh in 40 mL PBS. After centrifugation (50 *g* at 4 °C for 5 min), the supernatant was collected, followed by centrifugation at 2000 rpm at 4 °C for 10 min. The cell pellet was then resuspended with 5 mL of 40% Percoll (17-5445-01, Cytiva, USA) and centrifuged at 860 *g* at 4 °C for 15 min. Then, the red blood cells were lysed with 1 mL red blood cell lysis buffer (420301, Biolegend, USA). After being washed twice with PBS, the cells were resuspended in 200 μL PBS and ready for flow cytometry analysis.

### Detection of immune cell subsets by flow cytometry

Intrahepatic leukocytes were incubated in staining buffer with 1:100 anti-mouse CD16/32 (101302, Biolegend, USA) at room temperature for 5 min, and then with the following antibodies at 4 °C for 30 min: 1:50 anti-mouse CD45 (561868, BD Biosciences, USA), 1:50 anti-mouse CD3 (100205, Biolegend, USA), 1:50 anti-mouse CD4 (553046, BD Biosciences, USA), 1:50 anti-mouse CD8a (561109, BD Biosciences, USA), 1:50 anti-mouse CD19 (115537, Biolegend, USA), 1:50 anti-mouse NK1.1 (108709, BD Biosciences, USA), 1:50 anti-mouse CD11b (101207, Biolegend, USA), 1:50 anti-mouse F4/80 (123115, Biolegend, USA), 1:50 anti-mouse Ly-6G (560602, BD Biosciences, USA), and 1:50 anti-mouse Ly-6C (128031, Biolegend, USA). After incubation, the cells were washed and suspended in 200 μL FACS buffer, and analyzed by flow cytometry (CytoFLEX, Beckman Coulter, USA). The flow cytometry data were analyzed by FlowJo software (v10.0.7r2, Treestar, USA). The flow cytometry gating strategies for the analysis of leukocyte subpopulations (B, T, NK, NKT, CD4^+^ T, CD8^+^ T, monocytes, and macrophages) in liver tissue samples are shown in Figure S1.

### Statistical analysis

All statistical analyses and graphing were performed using GraphPad Prism 8.0 (GraphPad Software Inc, USA) and SPSS 26.0 (SPSS Inc, USA). Data that were normally distributed with homogeneous variance were presented as mean ± standard error of the mean and analyzed using either a two-sided unpaired Student’s *t*-test or one-way ANOVA, followed by Tukey’s post hoc test for multiple comparisons. In cases of non-normal distribution and heterogeneous variance, the Mann–Whitney U test or Kruskal–Wallis test was applied, with Tukey’s post hoc test for multiple comparisons. Additionally, Ishak scores were statistically assessed using the rank-sum test. Statistical significance was defined as a two-tailed *p*-value of less than 0.05.

## Results

### Hepatic transcriptome profiles revealed significant down-regulation of mitochondrial OXPHOS genes at the early phase of HBV-related hepatic inflammation

**Figure 1:**
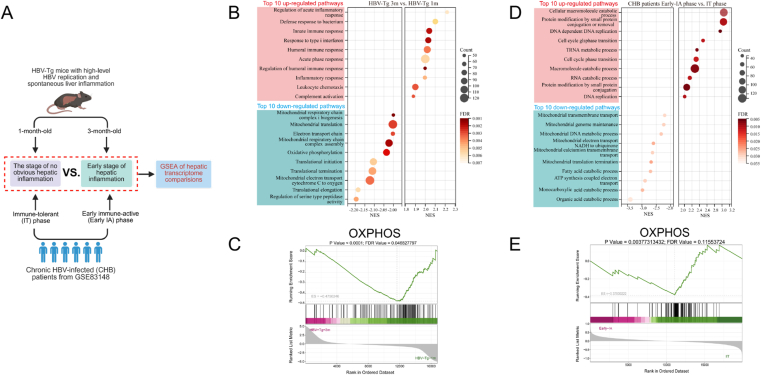
Significantly down-regulated mitochondrial OXPHOS pathways and gene expression at the early phase of HBV-induced hepatic inflammation. **(A)** Sketch map of hepatic transcriptome analysis. The hepatic transcriptome was compared between the early stage of hepatic inflammation and the stage of no obvious hepatic inflammation from HBV-Tg mice or CHB patients (public database GSE83148) by GSEA. **(B, D)** The bubble plots illustrate the top 10 significantly enriched up-regulated and down-regulated pathways in 3-month-old HBV-Tg mice versus 1-month-old HBV-Tg mice, as well as in CHB patients during the early-IA phase versus the IT phase. **(C, E)** The GSEA plot indicated down-regulated gene expression involved in mitochondrial OXPHOS in 3-month-old HBV-Tg mice versus 1-month-old HBV-Tg mice, or in CHB patients during the early-IA phase versus the IT phase. HBV-Tg, HBV transgenic mouse; CHB, chronic HBV hepatitis; GSEA, gene set enrichment analysis; OXPHOS, oxidative phosphorylation; IA, immune-active; IT, immune-tolerant; HBV, hepatitis B virus.

In our previous study, a unique early stage of HBV-induced hepatic inflammation, which was initiated by Stat3 activation, was identified in 3-month-old HBV-Tg mice and chronic HBV-infected patients (GSE83148) (further details and information can be found in our published article^11^). To explore the potential mechanism of Stat3 activation, we compared the hepatic transcriptome profiles of 1- and 3-month-old HBV-Tg mice, which were in high-level HBV replication with no evidence (1 month) or early signs (3 months) of hepatic inflammation (Fig. 1A). GSEA was performed using differentially expressed genes between 1-month-old HBV-Tg and 3-month-old HBV-Tg mice. The GSEA results demonstrated that the top ten up-regulated pathways in 3-month-old HBV-Tg mice were enriched in immune or inflammation-related pathways, including regulation of the acute inflammatory response, innate immune response, response to type I interferon, acute phase response, or leukocyte chemotaxis. The top ten down-regulated pathways in 3-month-old HBV-Tg mice were mainly linked to the mitochondrial OXPHOS function, such as the mitochondrial respiratory chain complex assembly, electron transport chain, OXPHOS, mitochondrial translation, or mitochondrial electron transport cytochrome C to oxygen (Fig. 1B). Furthermore, the GSEA enrichment plot and gene expression heatmap also revealed a remarkable down-regulation of oxidative phosphorylation genes, especially mitochondrial complex I and complex Ⅲ genes, in the 3-month-old mice group (Fig. 1C; Fig. S2A).

Meanwhile, we also analyzed liver transcriptomic signature differences between patients with CHB in the immune-tolerant phase and patients in the early immune-active phase. Similar to the liver transcriptomic characteristics of 3-month-old HBV-Tg mice, down-regulated mitochondrial OXPHOS-related pathways and genes were also found in patients with CHB during the early immune-active phase when compared with the immune-tolerant phase (Fig. 1D and E; Fig. S2B).

### HBV replication induced mitochondrial OXPHOS disorders and ROS accumulation

**Figure 2:**
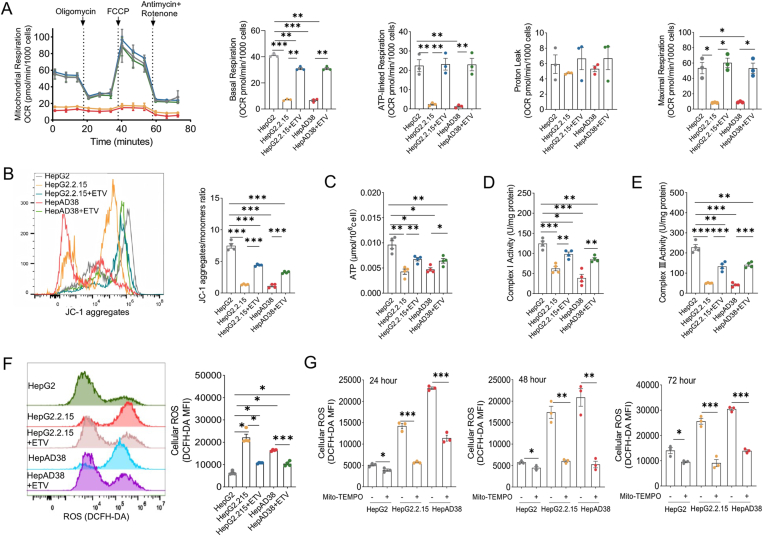
Mitochondrial OXPHOS dysfunction and excess ROS accumulation in HBV-replication hepatoma cell lines. **(A)** OCR was measured by Seahorse analyzer to determine basal respiration, ATP-linked respiration, proton leak, and maximal respiration capacity in HepG2 and HBV-replication hepatoma cell lines (HepG2.2.15 and HepAD38) with or without antiviral agent (ETV) treatment. **(B)** MMP was detected using JC-1 dye by flow cytometry in HepG2, HepG2.2.15, and HepAD38 with or without ETV treatment. A decrease in the JC-1 aggregates/monomers ratio indicated declined MMP, mitochondrial depolarization, or mitochondrial dysfunction. **(C**–**E)** Intracellular ATP levels and mitochondrial complex Ⅰ or Ⅲ activity were assayed in HepG2, HepG2.2.15, and HepAD38 with or without ETV treatment. **(F)** Cellular ROS levels were determined using DCFH-DA dye by flow cytometry in HepG2, HepG2.2.15, and HepAD38 with or without ETV treatment. Higher MFI of DCFH-DA dye indicated more ROS production in cells. **(G)** Cellular ROS levels were detected using DCFH-DA dye by flow cytometry in HepG2, HepG2.2.15, and HepAD38 cells with or without treatment of mitochondria-targeted ROS scavenger Mito-TEMPO after 24, 48, and 72 h of incubation period. All data were shown as mean ± standard error of the mean from at least 3 independent experiments. *P*-values < 0.05 were considered statistically significant (∗*P* < 0.05, ∗∗*P* < 0.01, or ∗∗∗*P* < 0.001 between two indicated groups). OCR, oxygen consumption rate; ETV, entecavir; MMP, mitochondrial membrane potential; ATP, adenosine triphosphate; ROS, reactive oxygen species; MFI, mean fluorescence intensity; HBV, hepatitis B virus.

The above analysis strongly suggested the potential role of down-regulated mitochondrial respiratory function in the development of HBV-induced hepatic inflammation. Therefore, in this part, we made a comprehensive assessment of the mitochondrial OXPHOS function in different HBV-replication cell lines: HepG2.2.15 and HepAD38. The oxygen consumption rates measured by the Seahorse Mito-stress test revealed a significant reduction of basal, maximal, and ATP-linked mitochondrial respiration in HepG2.2.15 and HepAD38 when compared with the HepG2 cells. Entecavir treatment could effectively inhibit HBV replication (Fig. S3) and improve the mitochondrial respiratory capacity of HepG2.2.15 or HepAD38 cells to be equivalent to HepG2 cells (Fig. 2A). Moreover, a significant decrease in the ratio of JC-1 aggregates to monomers was observed in HepG2.2.15 and HepAD38 cells, which could be reduced by entecavir treatment, indicating the disruption of normal mitochondrial membrane potential by HBV replication (Fig. 2B). Remarkably decreased intracellular ATP levels, another indicator of mitochondrial OXPHOS function, were also observed in HBV-replicating cells (Fig. 2C). Considering the crucial role of mitochondrial complexes I and III in the mitochondrial respiratory chain, their activities were also examined. HepG2.2.15 and HepAD38 cells showed a notable decline in mitochondrial complex I and III activity, which partially recovered after entecavir treatment (Fig. 2D and E). Furthermore, within the culture duration of 24–72 h, there was a gradual decrease in JC-1 aggregates, ATP levels, and mitochondrial complex I and III activity for HepG2.2.15, HepAD38, and HepG2 cells. However, the decreasing trend was more pronounced in the HepG2.2.15 and HepAD38 cells (Fig. S4A–S4D). Consistent with these findings of mitochondrial OXPHOS disorders, significantly increased cellular and mitochondrial ROS levels were also found in these HBV-replication cells, which could be remarkably diminished by entecavir treatment (Fig. 2F; Fig. S4E). Moreover, Mito-TEMPO, a targeted mitochondrial ROS scavenger, significantly reduced the cellular ROS levels in HepG2.2.15 and HepAD38 cells, indicating that enhanced ROS production mainly originated from the mitochondria (Fig. 2G).

### HBV-induced ROS accumulation enhanced activation of the Jak2/Stat3 signaling pathway

**Figure 3:**
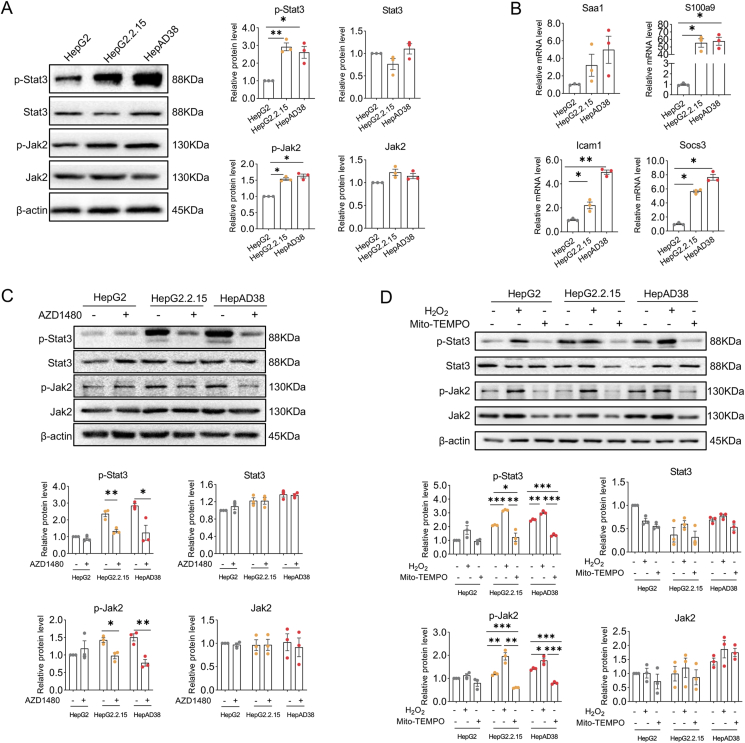
Increased Jak2/Stat3 activation by HBV replication-induced ROS accumulation. **(A)** The protein levels of p-Stat3, total Stat3, p-Jak2, and total Jak2 were examined by western blotting in HepG2, HepG2.2.15, and HepAD38 cell lines. **(B)** The mRNA expression of downstream target genes of p-Stat3 (Saa1, S100a9, Icam1, and Socs3) in HepG2, HepG2.2.15, and HepAD38 cell lines was detected by real-time quantitative PCR. **(C)** Inhibition of p-Jak2 activity by AZD1480 resulted in decreased protein levels of p-Stat3 and p-Jak2 in HepG2.2.15 and HepAD38 cells. **(D)** Effect of increasing or decreasing cellular ROS by exogenous H_2_O_2_ or Mito-TEMPO treatment on protein levels of p-Stat3, total Stat3, p-Jak2, and total Jak2 in HepG2, HepG2.2.15, and HepAD38 cells. The relative protein or mRNA expression was shown as fold-change in comparison with normal HepG2 cells (blank control). All data were shown as mean ± standard error of the mean from at least 3 independent experiments. *P*-values < 0.05 were considered statistically significant (∗*P* < 0.05, ∗∗*P* < 0.01, or ∗∗∗*P* < 0.001 between two indicated groups). Stat3, signal transducer and activator of transcription 3; p-Stat3, phosphorated Stat3; Jak2, Janus kinase 2; p-Jak2, phosphorated Jak2; Saa1, serum amyloid a1; S100a9, S100 calcium-binding protein A9; Icam1, intercellular adhesion molecule 1; Socs3, suppressor of cytokine signaling 3; H_2_O_2_, hydrogen peroxide; ROS, reactive oxygen species; HBV, hepatitis B virus.

Based on the results above showing that HBV replication could induce mitochondrial respiration dysfunction and ROS overproduction, in this section, the relationship between ROS overproduction and Stat3 activation was investigated. As we know, Stat3 is predominantly activated by Jak phosphorylation-dependent phosphorylation of tyrosine 705 (pY705).^19^ Given that no significant differences in the protein levels of phosphorylated Jak1 (p-Jak1) and p-Jak3 were observed among HepG2, HepG2.2.15, and HepAD38 cells in our preliminary experiments (data not shown), we focused on observing differences in the activation of the Jak2/Stat3 pathway here. Significantly higher protein levels of both phosphorylated Stat3 (p-Stat3 Y705) and Jak2 (p-Jak2 Y1007/1008) was found in HepG2.2.15 and HepAD38 cells than HepG2 cells, though without obvious changes in the expression of total Stat3 and Jak2 (Fig. 3A). Activation of the Stat3 signaling pathway was further confirmed by remarkably elevated mRNA expression of direct Stat3 target inflammatory genes (Saa1, S100a9, Icam1, and Socs3) (Fig. 3B). The reduction of p-Jak2 expression by p-Jak2 specific inhibitor (AZD1480) considerably suppressed the levels of p-Stat3, whereas without effect on expression of total Stat3 and Jak2 (Fig. 3C). Subsequently, the potential association between expression of p-Stat3 and p-Jak2 and ROS levels was explored by increasing intracellular ROS production (H_2_O_2_) or ROS scavenging (Mito-TEMPO). The levels of p-Stat3 and p-Jak2 were significantly up-regulated by H_2_O_2_ treatment, while significantly down-regulated by Mito-TEMPO treatment (Fig. 3D). Therefore, these findings suggested that ROS functioned as a crucial and upstream activator of the Jak2/Stat3 signaling pathway in HBV replication cells.

### Nrf2-induced cytokines communicated ROS to the activation of the Jak2/Stat3 pathway

**Figure 4:**
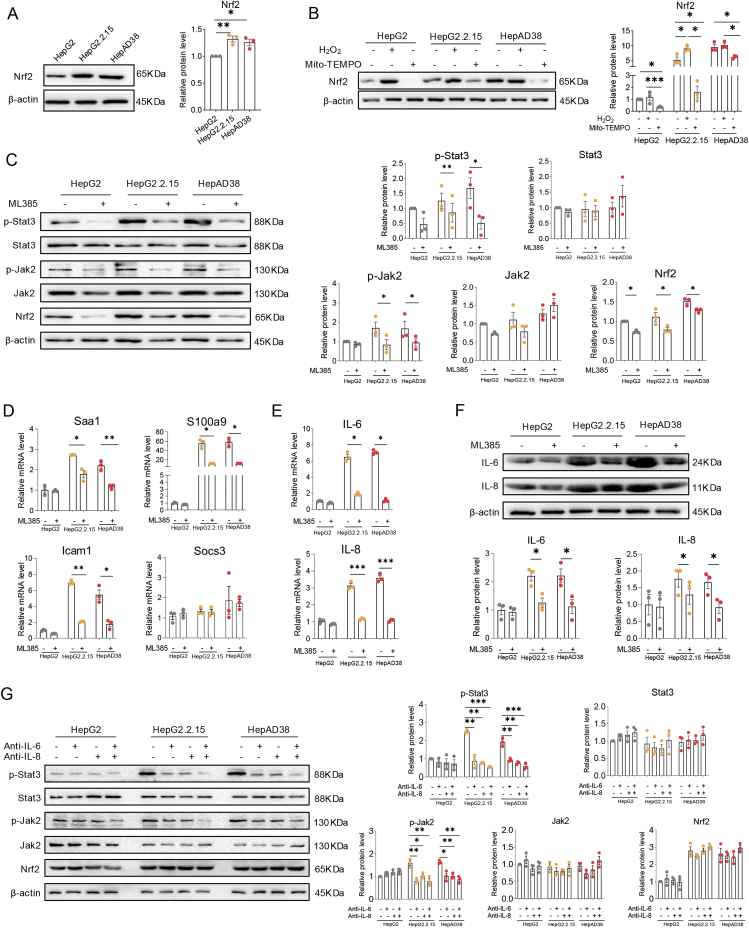
Nrf-2-associated IL-6 and IL-8 expression mediated ROS-induced Jak2/Stat3 activation. (**A)** Significantly increased protein expression of Nrf-2 in HepG2.2.15 and HepAD38 cell lines. **(B)** Protein expression levels of Nrf-2 were significantly increased after exogenous H_2_O_2_ treatment, or significantly decreased after Mito-TEMPO treatment. **(C)** Specific Nrf-2 inhibitor ML385 significantly reduced protein expression levels of Nrf-2, p-Stat3, and p-Jak2 in HepG2.2.15 and HepAD38 cells. **(D)** mRNA expression of downstream target genes of p-Stat3 (Saa1, S100a9, Icam1, and Socs3) was significantly suppressed after Nrf-2 inhibitor ML385 treatment. **(E)** Significant suppression of IL-6 and IL-8 mRNA expression in HepG2.2.15 and HepAD38 cells after Nrf-2 inhibitor ML385 treatment. **(F)** Significant suppression of IL-6 and IL-8 protein expression in HepG2.2.15 and HepAD38 cells after Nrf-2 inhibitor ML385 treatment. **(G)** The blockade of IL-6 and IL-8 signaling by anti-IL-6 or/and anti-IL-8 resulted in significantly decreased protein levels of p-Stat3 and p-Jak2 in HepG2.2.15 and HepAD38 cells. The relative protein or mRNA expression was shown as fold-change in comparison with normal HepG2 cells (blank control). All data were shown as mean ± standard error of the mean from at least 3 independent experiments. *P*-values < 0.05 were considered statistically significant (∗*P* < 0.05, ∗∗*P* < 0.01, or ∗∗∗*P* < 0.001 between two indicated groups). Nrf-2, nuclear factor erythroid 2-related factor 2; IL, interleukin; IFN, interferon; ROS, reactive oxygen species.

Subsequently, we next examined the mechanism by which ROS activated the Jak2/Stat3 pathway. Nrf2 has been shown to serve as the master regulator of cellular redox homeostasis, orchestrating the cellular response to oxidative stress.^20^ To investigate the role of Nrf2 in ROS-induced Stat3 phosphorylation, we first examined the protein expression level of Nrf2 in HBV replication cells. As shown in Figure 4A, Nrf2 protein levels were markedly elevated in Hep2.215 and HepAD38 cells compared with HepG2 cells (Fig. 4A). Meanwhile, the expression of Nrf2 protein displayed a significant increase after H_2_O_2_ treatment while a notable decrease after Mito-TEMPO treatment (Fig. 4B). Furthermore, the addition of ML385 (an Nrf2 inhibitor) to the culture medium resulted in a substantial reduction in the protein expression levels of p-Stat3 and p-Jak2 (Fig. 4C). Stat3 downstream target inflammation-related genes (Saa1, S100a9, and Icam1) exhibited obvious decreased mRNA levels after ML385 treatment (Fig. 4D). Given that the Jak2/Stat3 signaling pathway is mainly regulated by cytokines, we further explored whether Nrf2 would induce cytokines and contribute to the activation of the Jak2/Stat3 pathway. We detected several Jak2/Stat3 activation-related inflammatory cytokines, including IL-6, IL-8, interferon-alpha (IFN-α), IFN-γ, and IL-10 (Fig. S5). Additionally, we found that the mRNA and protein expression of IL-6 and IL-8 were significantly up-regulated in Hep2.2.15 and HepAD38 cells and remarkably decreased after treatment with Nrf2 inhibitor (Fig. 4E and F; Fig. S5). Inhibition of IL-6 and IL-8 signaling using blocking antibody of IL-6 or IL-8 in the culture medium significantly reduced the levels of p-Jak2 and p-Stat3. Furthermore, a combination treatment of IL-6 and IL-8 antibodies enhanced these inhibitory effects (Fig. 4G).

### Enhanced ROS levels with concurrent increased hepatic inflammation and activation of the Jak2/Stat3 pathway in HBV replication mice

**Figure 5:**
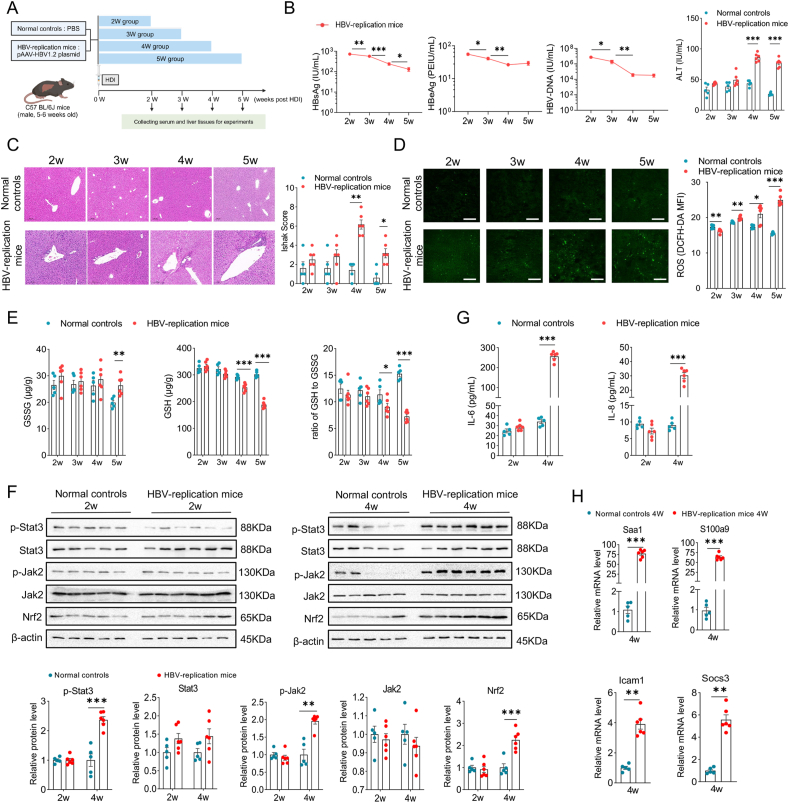
Hepatic ROS levels and Jak2/Stat3 activity were increased simultaneously at the early stage of liver inflammation in a persistent HBV-replication mouse model. **(A)** Schematic representation of experiment design and procedures. A persistent HBV replication mouse model was established by HDI of 6 μg pAAV-HBV 1.2 plasmid into 5 to 6-week-old male C57BL/6J mice. As normal controls, mice were injected hydrodynamically with the same volume of phosphate-buffered saline (PBS). Serum and liver samples from HBV-replication mice and normal controls were collected at 2, 3, 4, and 5 weeks after HDI for later experiments. **(B)** Dynamic changes in serum ALT levels of HBV-replication mice and normal controls collected at 2, 3, 4, and 5 weeks after HDI, or HBsAg, HBeAg, and HBV-DNA levels in HBV-replication mice. **(C)** Representative images of hematoxylin-eosin staining of liver tissue sections from normal controls (top row) and HBV-replication mice (bottom row). Ishak scoring system was used to evaluate the degrees of liver inflammation and fibrosis. **(D)** Detection of hepatic ROS on frozen section of liver tissue using DCFH-DA dye by fluorescence confocal microscope. The ROS levels were expressed as MFI of DCFH-DA dye and compared among these groups. **(E)** Measurement of GSH or GSSG in fresh liver tissues obtained from HBV-replication mice or controls. Reduced ratio of GSH to GSSG indicated increased oxidative stress. **(F)** Protein expression of p-Stat3, p-Jak2, Nrf2, Stat3, and Jak2 in liver tissues from the 2-week group (normal ALT levels and no obvious liver inflammation) and the 4-week group (increased ALT levels and early stage of significant liver inflammation) of HBV-replication mice and their controls. The relative protein expression was shown as fold-change in comparison with the 2-week group of controls. **(G)** Serum levels of IL-6 and IL-8 in the 2-week and 4-week groups of HBV-replication mice and their controls were assessed by ELISA. **(H)** Relative mRNA expression of downstream target genes of p-Stat3 (Saa1, S100a9, Icam1, and Socs3) in liver tissues from the 4-week group of HBV-replication mice and their controls was detected by real-time PCR. All data were shown as mean ± standard error of the mean. Each dot represented an individual mouse, and each group consisted of 5 or 6 mice. *P*-values < 0.05 were considered statistically significant (∗*P* < 0.05, ∗∗*P* < 0.01, or ∗∗∗*P* < 0.001 between two indicated groups). HDI, hydrodynamic injection; MFI, mean fluorescence intensity; GSH, reduced glutathione; GSSG, oxidized glutathione; ROS, reactive oxygen species; HBV, hepatitis B virus.

To determine if ROS-mediated Jak2/Stat3 activation were also observed *in vivo*, the pAAV/HBV1.2 mouse model with persistent HBV replication was used to evaluate the changes in the hepatic levels of ROS, inflammatory molecules, and activation of Jak2/Stat3 signaling (Fig. 5A). These HBV-replication mice remained constantly positive for serum HBV-DNA, HBsAg, and HBeAg till 5 weeks post hydrodynamic injection of the pAAV/HBV1.2 plasmid (Fig. 5B). The severity of liver inflammation was dynamically evaluated by liver histopathology scoring (Ishak score), ALT levels, and percentages of liver-infiltrating inflammatory cells. Compared with the individual control group, similar ALT levels and Ishak scores were observed in the pAAV/HBV1.2 mice at 2- or 3 weeks post-injection, while significantly elevated ALT levels and Ishak scores were found at 4- and 5 weeks post-injection (Fig. 5B and C). Meanwhile, intrahepatic leukocyte subsets were dynamically assessed by flow cytometry. Significantly higher percentages of liver CD19^+^ B cells, CD3^+^ T cells, NK cells (CD3^–^NK1.1^+^), pro-inflammatory monocytes (CD11b^+^Ly6C^high^), and monocyte-derived macrophages (F4/80^+^CD11b^+^Ly6C^low/high^) were also found in pAAV/HBV1.2 mice at 4 or 5weeks post-injection (Fig. S6).

Subsequently, hepatic oxidative stress was assessed by ROS levels and GSH/GSSG ratio. Compared with the control group, significantly increased ROS levels were exhibited in the liver tissues from pAAV/HBV1.2 mice at 3, 4, and 5 weeks post-injection, while a notable reduced ratio of GSH/GSSG could only be observed at 4 and 5 weeks post-injection (Fig. 5D and E). Next, we detected the expression of Nrf2 and activity of Jak2/Stat3 pathway in the liver tissues from pAAV/HBV1.2 mice at 2 weeks (no liver inflammation) or 4 weeks (significant liver inflammation) post-injection. As shown in Figure 5F, a remarkable elevation in protein expression of p-Stat3, p-Jak2, and Nrf2 was found in pAAV/HBV1.2 mice at 4 weeks post-injection compared with the control group, while no obvious changes were found at 2 weeks. Moreover, higher levels of serum IL-6 and IL-8 and enhanced mRNA expression of Stat3 target genes (such as Saa1, S100a9, Icam1, and Socs3) were further observed in pAAV/HBV1.2 mice at 4 weeks post-injection (Fig. 5G and H).

### Scavenging ROS hindered activation of the Jak2/Stat3 pathway and alleviated hepatic inflammation *in vivo*

**Figure 6:**
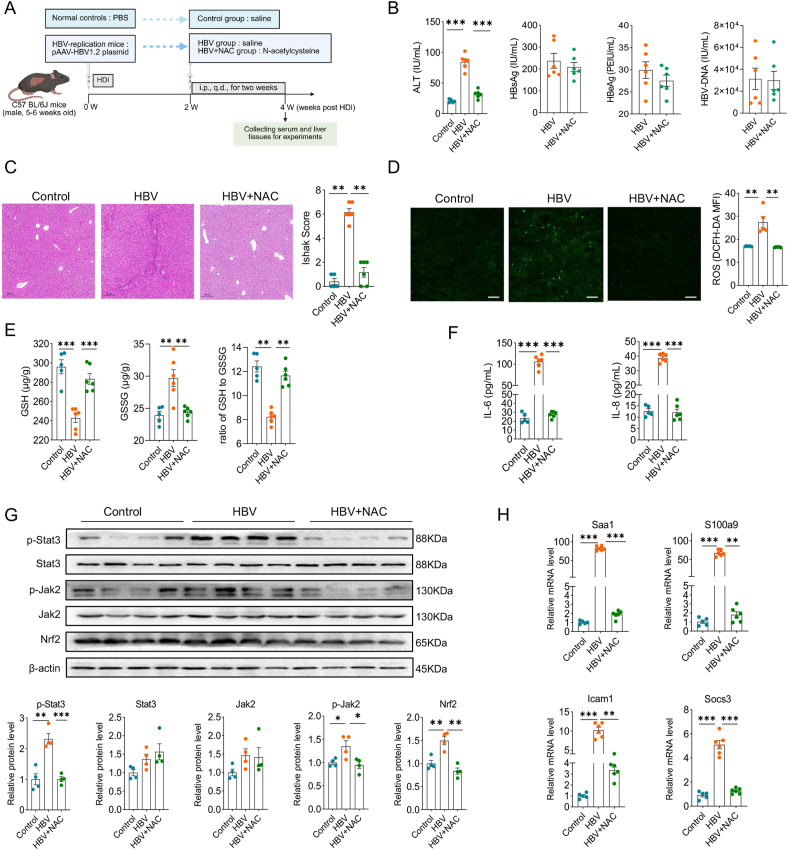
Scavenging ROS by NAC suppressed the Jak2/Stat3 pathway activation and attenuated liver inflammation in HBV-replication mice. **(A)** Experiment procedure flowchart. The HBV-replication mice were established by HDI of 6 μg pAAV-HBV 1.2 plasmid, while the mice of normal controls were injected with phosphate-buffered saline (PBS) in the same volume. Two weeks after HDI, HBV-replication mice were injected intraperitoneally every day with either NAC (300 mg/kg at 200 μL saline) (HBV + NAC group) or saline solution (HBV group) for another 2 weeks. Four weeks after HDI, mice of the control, HBV + NAC, and HBV groups were sacrificed, and serum and liver tissue samples were collected for experiments. **(B, C)** NAC treatment effectively decreased serum ALT levels and the degree of liver inflammation (shown as Ishak Score) in the HBV + NAC group compared with the HBV group. **(D)** Significantly reduced hepatic ROS levels in the HBV + NAC group compared with the HBV group. ROS levels on frozen section of liver tissue were detected using DCFH-DA dye by fluorescence confocal microscope and showed as MFI of DCFH-DA dye. **(E)** Remarkably higher GSH content, lower GSSG content, and a higher ratio of GSH to GSSG in the HBV + NAC group than in the HBV group. **(F)** Serum IL-6 and IL-8 levels were significantly decreased in the HBV + NAC group compared with the HBV group. **(G)** NAC treatment significantly inhibited protein expression of Nrf-2, p-Jak2, and p-Stat3 in liver tissues from the HBV + NAC group compared with the HBV group. The relative protein expression was shown as fold-change in comparison with the control group. **(H)** NAC treatment significantly inhibited mRNA expression of downstream target genes of p-Stat3 (Saa1, S100a9, Icam1, and Socs3) in liver tissues from the HBV + NAC group compared with the HBV group. The relative mRNA expression was shown as fold-change in comparison with the control group. All data were shown as mean ± standard error of the mean. Each dot represented an individual mouse, and each group consisted of 5 or 6 mice. *P*-values < 0.05 were considered statistically significant (∗*P* < 0.05, ∗∗*P* < 0.01, or ∗∗∗*P* < 0.001 between two indicated groups). NAC, N-acetylcysteine; HDI, hydrodynamic injection; MFI, mean fluorescence intensity; GSH, reduced glutathione; GSSG, oxidized glutathione; ROS, reactive oxygen species; HBV, hepatitis B virus.

In this section, NAC, one of the most widely used antioxidants, was applied to verify the relationship between ROS and liver inflammation in HBV replication mice (Fig. 6A). Compared with pAAV/HBV1.2 mice with saline treatment (HBV group), HBV mice with NAC treatment (HBV + NAC group) exhibited significantly reduced ALT levels, while no notable changes in HBsAg, HBeAg, or HBV-DNA levels were observed (Fig. 6B). Furthermore, liver tissues from the HBV + NAC group had obviously lower Ishak scores and lower percentages of intrahepatic NK cells, NKT cells, CD3^+^ T cells, monocytes, and monocyte-derived macrophages than the HBV group (Fig. 6C; Fig. S7). ROS fluorescence staining of liver tissue sections reflected a noticeable decrease of ROS in the HBV + NAC group (Fig. 6D). In addition, hepatic oxidative stress was effectively alleviated in pAAV/HBV1.2 mice by NAC treatment, evidenced by increased GSH and decreased GSSG (Fig. 6E). ELISA results indicated a significant reduction in IL-6 and IL-8 in the serum of HBV + NAC mice (Fig. 6F). Furthermore, NAC treatment remarkably reduced the protein expression of p-Stat3, p-Jak2, and Nrf2, and mRNA levels of Stat3 target genes (Saa1, S100a9, Icam1, and Socs3) in the liver of pAAV/HBV1.2 mice (Fig. 6G and H).

## Discussion

Accumulating studies have demonstrated the significant roles of liver inflammation in the progression of CHB to liver cirrhosis and hepatocarcinoma.^21^ However, few studies have focused on the early or initial phase of liver inflammation in chronic HBV infection. Thus, currently, little is known about the mechanisms triggering the transition from the immune-tolerant phase without liver inflammation to the immune-active phase with significant liver inflammation. Difficulties in the identification of the early phase of liver inflammation by serological markers of liver function in patients with CHB, usually with normal ALT levels at this phase,^22^ are the main obstacle to exploring this mechanism.

Therefore, in our previous study, an HBV-replicating mouse model (HBV-Tg mouse) with spontaneous liver inflammation was applied to dynamically and continuously monitor the occurrence of hepatic inflammation with serum ALT, serum and hepatic HBV markers, liver histology, and hepatic transcriptome. By this method, we successfully identified the early phase of HBV-induced liver inflammation, which was characterized by normal serum ALT levels, mild liver inflammation, and marked up-regulation of hepatic genes related to inflammation, innate immunity, and chemotaxis of cells.^11^

To explore the mechanism underlying the occurrence of hepatic inflammation, the features of the hepatic transcriptome at the early phase of liver inflammation were comprehensively analyzed. In addition, our previous results revealed the triggering role of Stat3 in the occurrence of inflammation.^11^ These observations were further verified by an analysis of the public human CHB dataset. However, the upstream events of Stat3 activation still need to be elucidated.

**Figure 7:**
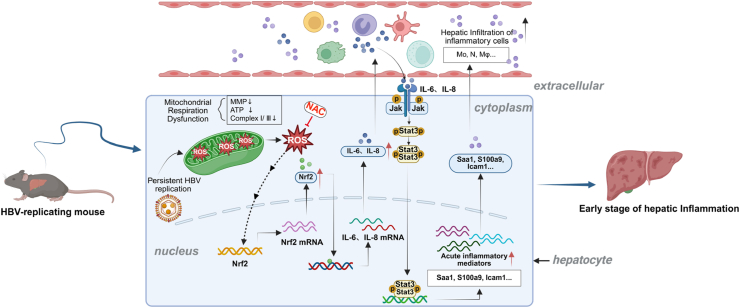
Schematic overview of proposed mechanisms. Persistent HBV replication in hepatocytes may cause mitochondrial respiration dysfunction and further result in ROS accumulation. In response to elevated ROS levels, protein expression of Nrf-2 was increased, leading to transcriptional up-regulation of cytokines IL-6 and IL-8. Autocrine/paracrine IL-6 and IL-8 bound to their membrane receptors that recruited and activated Jak2. Then, Stat3 was recruited and subsequently phosphorylated by Jak2. Upon phosphorylation, p-Stat3 underwent homodimerization, allowing for translocation into the nucleus to promote transcriptional activation of a set of inflammatory genes, such as Saa1, S100a9, or Icam1. The surge of these inflammatory mediators caused sharply increased intrahepatic infiltration of immune and inflammatory cells, and then triggered the occurrence of liver inflammation. ROS scavenger N-NAC could decrease ROS production and suppress the process of ROS-triggered hepatic inflammation. NAC, N-acetylcysteine; ROS, reactive oxygen species; HBV, hepatitis B virus.

In this study, using HBV-replicating cell lines and a mouse model, we extend our previous findings by demonstrating that HBV-induced ROS accumulation enhanced Stat3 activation sharply via Nrf2-IL-6/IL-8-Jak2 signal cascades and played a pivotal role in liver inflammation occurrence (Fig. 7). An HBV-replicating mouse model used here was constructed by hydrodynamic injection with the pAAV/HBV1.2 plasmid.^23^ This mouse model was first reported by Huang et al in 2006, and has been proven to establish persistently HBV replication for more than three months.^24^ Compared with HBV transgenic (HBV-Tg) mice with persistent high levels of HBV DNA and antigens, while without HBV-specific immunity, pAAV/HBV1.2 mice were immunocompetent and therefore widely used for investigating both the efficacy of antiviral drugs and HBV-related immune responses.^25^ We unveiled an association between mitochondrial dysfunction and hepatic inflammatory infiltration in both HBV-Tg mice and pAAV/HBV1.2 mice. These findings hint that mitochondrial dysfunction-induced liver inflammation during chronic HBV infection might be independent of HBV-specific immune responses.

It has been thought that immaturity of the neonatal immune system, especially low or absent virus-specific T-cell responses, contributes to the development of the immune-tolerant phase of chronic HBV infection. It was also traditionally thought that the immunologic events occurring during the immune-active phase are related to the increasing number and function of HBV-specific T cells.^5^^,^^26^ This traditional concept is strongly challenged by the clinical and experimental evidence that the ALT level, reflecting the severity of hepatic inflammation, is not parallel to the level of HBV-specific T cells and cannot be used as a surrogate of HBV-specific T-cell activity.^6^^,^^7^^,^^27^^,^^28^ Actually, significant hepatic inflammation and ALT elevations during chronic HBV infection are proportional to the intrahepatic non-specific immune and inflammatory cells, including granulocytes, monocytes, and T cells. Therefore, an alternative hypothesis of the key differences in immune events between the immune-tolerant and immune-active phases is the heightened inflammatory events during the immune-active phase.^8^^,^^29^ However, the trigger factors of these inflammatory events are still unknown. Our results not only depicted a comprehensive profile of inflammatory response genes in the early immune-active phase, but also elucidated the trigger factors of these inflammatory events. Thus, our findings provide direct evidence showing that the non-specific inflammatory response characterized the immune/inflammatory events of the early immune-active phase, and hepatocellular mitochondrial dysfunction-initiated Stat3 activation triggered these inflammatory events.

In recent years, increasing attention has been paid to the impacts of HBV infection on mitochondrial function. Most research has been focused on the HBx protein, a multifunctional regulatory factor of HBV, which is identified to be located in the inner and outer membrane, and the mitochondrial matrix. These results showed that HBx could interact directly with several mitochondrial-related proteins and affect various intracellular inflammation, apoptosis, and immunity signaling pathways, such as activating Stat3 and NF-κB, through ROS level elevation, up-regulating cytoplasmic calcium with control of the voltage-dependent anion channel component, or suppressing the mitochondrial antiviral signaling (MAVS)-mediated IFN-β induction by ubiquitinating MAVS.^16^^,^^30^, ^31^, ^32^, ^33^ For the studies based on the patients with chronic HBV infection, significantly decreased mitochondrial function, mitochondrial DNA content, mitophagy, or mitochondrial biogenesis were also observed in liver tissues showing advanced liver fibrosis, cirrhosis, or hepatocarcinoma.^17^^,^^18^ These results indicated that chronic HBV infection might mediate mitochondrial dysfunction and promote the development and progression of liver fibrosis or advanced liver diseases. Our research focused on the mechanism of inflammation occurrence in the early stages of chronic HBV infection or in the transition from the immune-tolerant to immune-active phase, and found the key role of ROS-related Stat3 activation in this mechanism. Therefore, our findings enriched existing research on the roles of HBV-induced mitochondrial dysfunction in chronic HBV infection and disease progression. Furthermore, our study also hinted at the different roles of HBV-related mitochondrial dysfunction-induced liver inflammation in different stages of chronic HBV infection, which might be a “good” regulator of anti-HBV immune responses and viral clearance in the early stage, while it might be a “bad” factor of chronic inflammation and disease progression in the late stage.

Mitochondrial dysfunction has been verified to be closely related to the inflammatory response involved in numerous physiological processes and disorders.^34^, ^35^, ^36^ Various mitochondrial components and products (such as mtRNA, mtDNA, ROS, or ATP) can be released into the cytosol as a consequence of mitochondrial dysfunction and then activate several inflammatory signaling pathways, including cGAS-STING, NF-κB, and inflammasome pathways.^37^, ^38^, ^39^ Among these mitochondrial-derived inflammatory components, ROS has been reported to be central to the pathogenesis of several inflammatory diseases.^40^ For viral diseases, dysregulated ROS formation has been proven to serve as a crucial contributor to inflammatory surge in the pathogenesis of virus infection, especially for RNA viral infections, such as the influenza virus or coronavirus.^41^, ^42^, ^43^ Here, our results highlighted that ROS overproduction also played a key role in triggering the inflammatory responses during chronic HBV infection, and suggested a new perspective to get a deeper understanding of the mechanism of inflammation occurrence during persistent viral infections.

There were some limitations in our study. Firstly, our findings would benefit from further validation through the examination of liver tissues from patients with CHB. Secondly, it remains unclear whether ROS has an impact on the infiltration of HBV-specific immune cells. Lastly, additional investigation is required to understand why HBV appears to have a more pronounced effect on mitochondrial electron transport chain complexes I and III.

Collectively, our research demonstrates that HBV-induced ROS enhances Stat3 activation, playing a pivotal role in triggering HBV-related liver inflammation, offering new insights into the key factor triggering hepatic inflammation during chronic HBV infection.

## CRediT authorship contribution statement

**Rui Song:** Writing – review & editing, Writing – original draft, Validation, Methodology, Data curation. **Shasha Yu:** Writing – review & editing, Writing – original draft, Visualization, Validation, Conceptualization. **Xueyan Chen:** Validation, Investigation, Data curation. **Ning Ling:** Supervision, Resources, Formal analysis. **Dachuan Cai:** Supervision, Project administration, Funding acquisition, Conceptualization. **Hong Ren:** Supervision, Project administration, Funding acquisition, Conceptualization. **Min Chen:** Writing – review & editing, Writing – original draft, Supervision, Project administration, Methodology, Funding acquisition, Conceptualization.

## Funding

This work was supported by the Chongqing Medical Scientific Research Project (Joint Project of Chongqing Health Commission and Science and Technology Bureau) (China) (No. 2024MSXM024 to M.C.). This work was also supported by the Remarkable Innovation–Clinical Research Project, The Second Affiliated Hospital of 10.13039/501100004374Chongqing Medical University (2022) (to H.R.), and The First batch of key Disciplines on Public Health in Chongqing, Health Commission of Chongqing, China (to D.C.).

## Conflict of interests

The authors declared no conflict of interests.

## Figures and Tables

**Figure:**
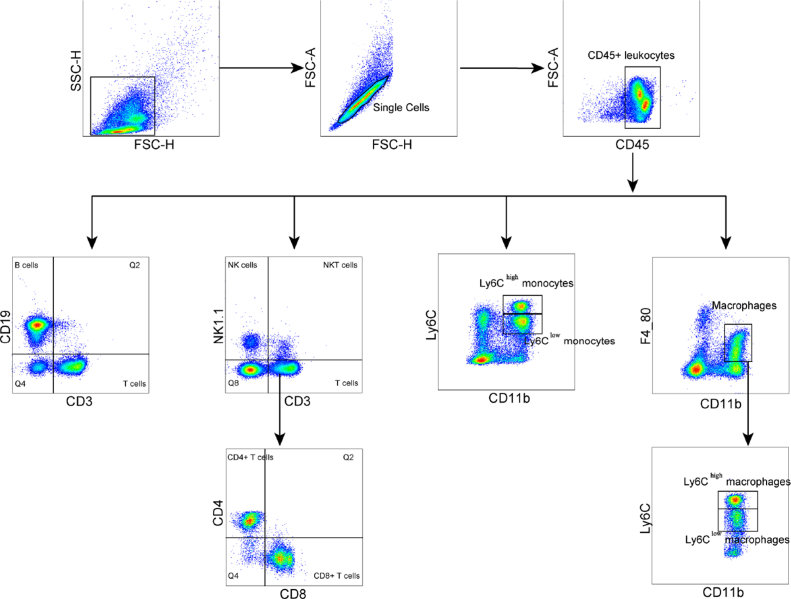


**Figure:**
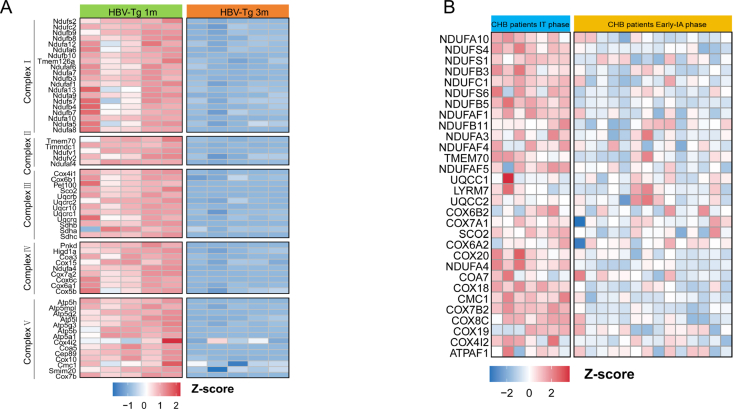


**Figure:**
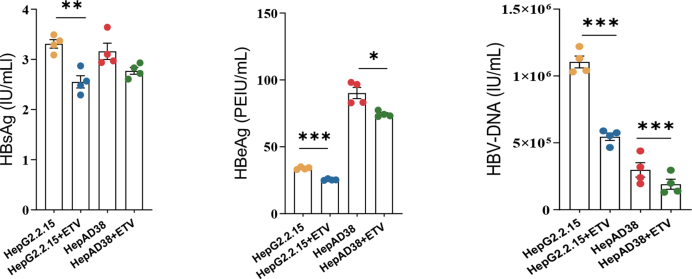


**Figure:**
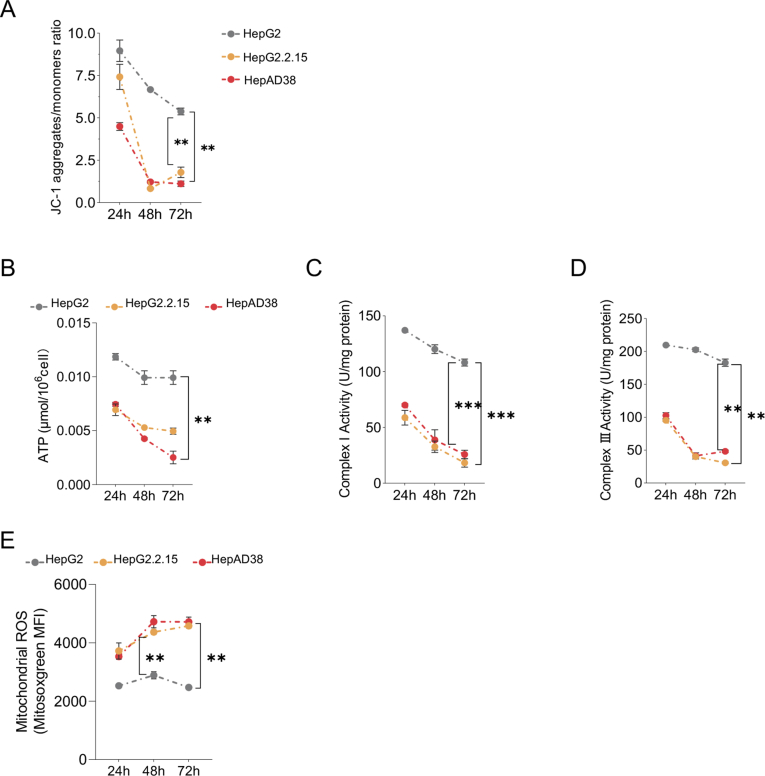


**Figure:**
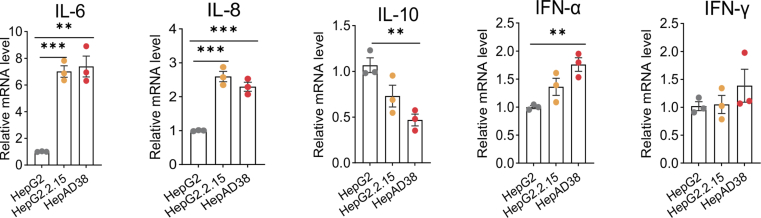


**Figure:**
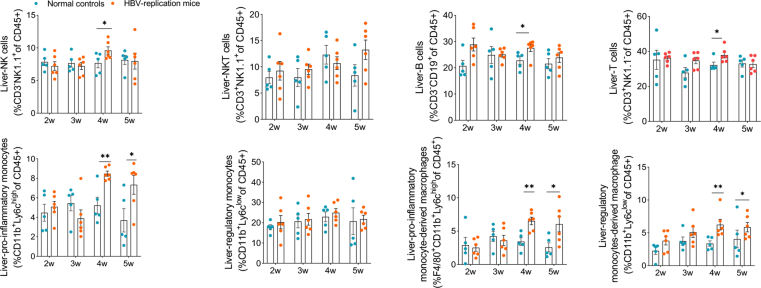


**Figure:**