# Supplementary material for: Activation of the Jak2/Stat3 pathway by ROS-dependent signaling cascades initiates hepatitis B virus-induced hepatic inflammatory responses
Source: Genes Dis. 2025 Sep 23;13(5):101857. doi: 10.1016/j.gendis.2025.101857 (PMC13254580; doi:10.1016/j.gendis.2025.101857)
Supplement: Multimedia component 1 [file mmc1.docx]

**Figure S1** Flow cytometry gating strategies for analyzing intrahepatic leukocytes.

**Figure S2** Heatmap for the significantly down-regulated mitochondrial OXPHOS genes in hepatic transcriptome from 3-month-old HBV-Tg mice compared with 1-month-old HBV-Tg mice (A), or CHB patients during the early-IA phase versus IT phase (B). Gene expression was log2 transformed and normalized to 1-month-old HBV-Tg mice or the CHB IT phase, respectively. IA, immune-active; IT, immune-tolerant.

**Figure S3** The levels of HBsAg, HBeAg, and HBV-DNA in the supernatant of HBV-replication cell lines HepG2.2.15 and HepAD38 treated with or without antiviral drugs (entecavir, ETV). All data were shown as mean ± standard error of the mean from at least 3 independent experiments. *P*-values <0.05 were considered statistically significant (^*^*P* < 0.05, ^**^*P* < 0.01, or ^***^*P* < 0.001 between two indicated groups).

**Figure S4** Dynamic changes in mitochondrial OXPHOS function in HepG2, HepG2.2.15, and HepAD38 cell lines after 24, 48, and 72 h of culture incubation. **(A)** Mitochondrial membrane potential (MMP) was detected using JC-1 dye by flow cytometry. A decrease in the JC-1 aggregates/monomers ratio indicated declined MMP, mitochondrial depolarization, or mitochondrial dysfunction. **(B–D)** Assay of intracellular ATP levels (B), complex Ⅰ activity (C), or complex Ⅲ activity (D). **(E)** Mitochondrial-specific ROS levels were evaluated with MFI of MitoSOX Green dye by flow cytometry assay. All data were shown as mean ± standard error of the mean from at least 3 independent experiments. *P*-values <0.05 were considered statistically significant (^*^*P* < 0.05, ^**^*P* < 0.01, or ^***^*P* < 0.001 between two indicated groups). ATP, adenosine triphosphate; ROS, reactive oxygen species; MFI, mean fluorescence intensity.

**Figure S5** Relative mRNA levels of potential Jak2/Stat3 activation-related inflammatory cytokines (IL-6, IL-8, IL-10, IFN-α, and IFN-γ) in HepG2, HepG2.2.15, and HepAD38 cell lines. Relative mRNA levels were displayed as the fold change compared with HepG2 cells. All data were shown as mean ± standard error of the mean from at least 3 independent experiments. *P*-values <0.05 were considered statistically significant (^*^*P* < 0.05, ^**^*P* < 0.01, or ^***^*P* < 0.001 between two indicated groups). IL, Interleukin; IFN, Interferon.

**Figure S6** Longitudinal analysis of percentages of intrahepatic leukocytes (NK, NKT, B, T, monocyte, and macrophage) isolated from the 2-week, 3-week, 4-week, and 5-week groups of HBV-replication mice or normal controls by flow cytometry. All data were shown as mean ± standard error of the mean from at least five mice per group. *P*-values <0.05 were considered statistically significant (^*^*P* < 0.05, ^**^*P* < 0.01, or ^***^*P* < 0.001 between two indicated groups).

**Figure S7** Analysis of percentages of intrahepatic leukocytes (NK, NKT, B, T, monocyte, and macrophage) in normal controls (control group) and HBV-replication mice treated with or without N-acetylcysteine (NAC) (HBV or HBV+NAC group) by flow cytometry. All data were shown as mean ± standard error of the mean from at least five mice per group. *P*-values <0.05 were considered statistically significant (^*^*P* < 0.05, ^**^*P* < 0.01, or ^***^*P* < 0.001 between two indicated groups).
